# Role of inhaled antibiotics in the era of highly effective CFTR modulators

**DOI:** 10.1183/16000617.0154-2022

**Published:** 2023-01-11

**Authors:** J. Stuart Elborn, Francesco Blasi, Pierre-Régis Burgel, Daniel Peckham

**Affiliations:** 1 Faculty of Medicine Health and Life Sciences, Queen's University, Belfast, UK; 2 Department of Pathophysiology and Transplantation, University of Milan, Milan, Italy; 3 Internal Medicine Department, Respiratory Unit and Cystic Fibrosis Adult Center, Fondazione IRCCS Ca’ Granda Ospedale Maggiore Policlinico, Milan, Italy; 4 Université Paris Cité, Institut Cochin, Paris, France; 5 Respiratory Medicine and Cystic Fibrosis National Reference Center, Cochin Hospital, Assistance Publique Hôpitaux de Paris (AP-HP), Paris, France; 6 Respiratory Medicine, Leeds Institute of Medical Research, University of Leeds, Leeds, UK

## Abstract

Recurrent and chronic bacterial infections are common in people with cystic fibrosis (CF) and contribute to lung function decline. Antibiotics are the mainstay in the treatment of exacerbations and chronic bacterial infection in CF. Inhaled antibiotics are effective in treating chronic respiratory bacterial infections and eradicating *Pseudomonas aeruginosa* from the respiratory tract, with limited systemic adverse effects. In the past decade, highly effective cystic fibrosis transmembrane conductance regulator (CFTR) modulators have become a new therapy that partially corrects/opens chloride transport in patients with selected CFTR mutations, restoring mucus hydration and improving mucociliary clearance. The recent triple CFTR modulator combination is approved for ∼80–90% of the CF population and significantly reduces pulmonary exacerbations and improves respiratory symptoms and lung function. CFTR modulators have shifted the focus from symptomatic treatment to personalised/precision medicine by targeting genotype-specific CFTR defects. While these are highly effective, they do not fully normalise lung physiology, stop inflammation or resolve chronic lung damage, such as bronchiectasis. The impact of these new drugs on lung health is likely to change the future management of chronic pulmonary infections in people with CF. This article reviews the role of inhaled antibiotics in the era of CFTR modulators.

## Abstract 2

**Studies are needed to determine whether discontinuing maintenance therapy is safe in people with cystic fibrosis receiving CFTR modulators. Until then, people with CF are recommended to continue their prescribed medications, including antibiotic therapy.** https://bit.ly/3ML3nYw

## Introduction

Cystic fibrosis (CF) is a disease that affects multiple organs, including the lungs, pancreas and gastrointestinal tract. Its clinical presentation is variable and includes recurrent and chronic respiratory infections, pancreatic insufficiency, malnutrition and male infertility [1, 2]. Acute and chronic respiratory infections and progressive lung disease remain the leading cause of morbidity and mortality [1]. Repeated episodic infective exacerbations in people with CF drive local and systemic inflammation, lung damage and decline in lung function [3].

Airway bacterial infections are very strongly associated with exacerbations, poor quality of life and reduced survival in people with CF [3]. *Pseudomonas aeruginosa*, other nonfermenting Gram-negative bacteria and nontuberculous mycobacteria are strongly associated with exacerbations in adolescents and adults, while *Haemophilus influenzae* and *Staphylococcus aureus* are more common in children and can be associated with exacerbations [4, 5]. Antibiotics are evidence-based proven therapies for the treatment of acute and chronic lung microbial infections in CF and are universally recommended in care guidelines for people with CF [6] (table 1). Although respiratory exacerbations are most often treated with systemic (oral and/or intravenous) antibiotics, inhaled therapy is the preferred route for long-term suppressive therapy. This allows the delivery of high drug concentrations directly to the airways, which can improve drug effectiveness, enhance the ease of use and limit systemic adverse effects. This makes inhaled therapy particularly effective as eradication therapy for *P. aeruginosa* and new microbial isolates, suppressing chronic endobronchial infections, treating pulmonary exacerbations, preventing infection-associated exacerbations of airway disease and thereby improving quality of life in patients with CF (table 2) [19]. Off-label use of intravenous antibiotics such as meropenem, vancomycin and amikacin are also used for inhalation in people with CF. The evidence to support this practice is minimal and beyond the scope of this article [20].

**TABLE 1:** Inhaled antibiotics approved for use in patients with cystic fibrosis

**Aztreonam lysine**	Effective against most Gram-negative bacteria including *Pseudomonas*; has activity against aminoglycoside-resistant *Pseudomonas aeruginosa*.
**Colistin**	Active only against certain Gram-negative aerobes and Gram-negative facultative anaerobes. Species that are susceptible to colistin include *Citrobacter* species*, Escherichia coli*, *Haemophilus influenzae* and *P. aeruginosa*.
**Levofloxacin^#^**	Broad spectrum of activity against Gram-positive and Gram-negative bacteria and atypical respiratory pathogens. It is active against both penicillin-susceptible and penicillin-resistant *Streptococcus pneumoniae*.
**Tobramycin**	Effective against *Pseudomonas* species, *Klebsiella* species, *E. coli* and both *Staphylococcus aureus* and *Staphylococcus albus*.

**^#^**: inhaled levofloxacin is not approved in France.

**TABLE 2:** Key studies of inhaled aztreonam, colistin and tobramycin conducted in people with cystic fibrosis

**First author, year [reference]**	**Formulation, dosage**	**Duration, patient population**	**Key outcomes after treatment**
**Aztreonam**			
McCoy, 2008 [7]	AZLI, 75 mg twice daily or three times daily	28 days with 56 days of follow-up n=211 receiving inhaled tobramycin	Reduced exacerbation rates, improvement in lung function and respiratory symptoms
Retsch-Bogart, 2009 [8]	AZLI, 75 mg three times daily	28 days n=164	Improvement in lung function and quality-of-life scores, and reduced hospital days
Oermann, 2010 [9]	AZLI, 75 mg twice daily or three times daily	28 days on, 28 days off (up to 9 cycles) n=195	Improvements in lung function and respiratory symptoms in patients treated three times daily
**Colistin**			
Jensen, 1987 [10]	CSI, 1 million units twice daily	3 months n=40	Improvements in symptom scores and slower decline in lung function
Hodson, 2002 [11]	CSI or TSI, 80 mg twice daily (CSI) or 300 mg twice daily (TSI)	4 weeks n=115	Improvement in lung function with TSI and not CSI. Both decreased bacterial load
Schuster, 2013 [12]	CDP or TSI, 1.6 million units twice daily (CDP) or 300 mg twice daily (TSI)	28 days on, 28 days off (3 cycles) n=380	CDP was demonstrated noninferior to TSI, but primary end-point regarding lung function was not met
**Tobramycin**			
MacLusky, 1989 [13]	TSI, 80 mg three times daily	32 months n=27	Stability in pulmonary function observed in treatment group while control group showed decline
Smith, 1989 [14]	TSI, 600 mg three times daily	12 weeks n=22	Improvement in symptoms and decrease in bacterial density
Ramsey, 1993 [15]	TSI, 600 mg three times daily	12 weeks, 28 days on, 28 days off n=71	Improvement in pulmonary function
Ramsey, 1999 [16]	TSI, 300 mg twice daily	24 weeks (on/off every 28 days) n=520	Improvement in pulmonary function and reduced hospitalisations
Konstan, 2011 [17]	TIP or TSI, 112 mg twice daily or 300 mg twice daily	28 days on, 28 days off (3 cycles) n=517	Efficacy of TIP was comparable with TSI. Greater satisfaction was observed with inhalation powder

AZLI: aztreonam solution for inhalation; CSI: colistin solution for inhalation; TSI: tobramycin solution for inhalation; CDP: colistin dry powder; TIP: tobramycin inhalation powder. Reproduced and modified from [18].

In the past four decades, there has been a significant increase in survival among people with CF due to the adoption of a multidisciplinary approach to care, aggressive antibiotic therapy, newborn screening, nutrition, addressing extrapulmonary symptoms and the recent availability of cystic fibrosis transmembrane conductance regulator (CFTR) modulators in some countries. CFTR modulators have been developed over the past decade and are effective in ∼90% of people with CF [21]. This new form of precision/personalised medicine targets specific mutations in the *CFTR* gene, thereby improving the expression and function of CFTR across epithelial membranes [22]. As these therapies improve forced expiratory volume in 1 s (FEV_1_) and reduce exacerbations, it is highly probable that they will further improve survival [22]. While clinical trials have demonstrated the efficacy of CFTR modulators in people with CF [22]; their effect on bacterial infection and subsequent airway inflammation remains less clear. There is a dearth of information in the literature to guide the decision of whether antibiotics could be stopped in people with CF after receiving CFTR modulators. As a result, the general consensus is that antibiotics should not be discontinued in the majority of patients on CFTR modulators and that an evidence base is needed before clear guidelines can be given. In addition, it is important to recognise that many people with CF, despite feeling a lot better, will have persistent and often significant structural lung damage. Furthermore, the long-term natural history of CF-related lung disease post-treatment with CFTR modulators remains unknown [23]. In this review, we discuss the role of inhaled antibiotics in people with CF treated with CFTR modulators.

## CF and current treatment: CFTR modulators

The CFTR modulators are small molecules that enhance or restore epithelial chloride and bicarbonate ion transport in people with selected CF-causing mutations. CFTR modulators include potentiators, which increase the activity of CFTR on epithelial surfaces, and correctors, which improve the processing and trafficking of defective protein and increase the amount of mutated CFTR at the cell membrane [24]. In most cases, a combination of a CFTR potentiator and two CFTR correctors is used for patients with responsive mutations [21, 25]. Currently licensed modulators include ivacaftor, lumacaftor, tezacaftor, elexacaftor and their combinations (table 3) [28, 29].

**TABLE 3:** Cystic fibrosis transmembrane conductance regulator (CFTR) modulators approved by the European Medicines Agency; the common responsive mutations; and their effects on patients with cystic fibrosis (CF)

**CFTR modulator**	**Common responsive mutations**	**Effects in patients with CF**
**Ivacaftor**	G551D, G178R, S549N, S549R, G551S, G1244E, S1251N, S1255P, G1349D, R117H	Increase in lung function (FEV_1_%), reduction in frequency of pulmonary exacerbations, increase in weight and respiratory-related quality of life, decrease in days with intravenous antimicrobial treatment
**Lumacaftor/ivacaftor**	Phe508del-homozygous patients	Increase in lung function (FEV_1_%), reduction in hospitalisations, reduction in intravenous antibiotics use and pulmonary exacerbations, increase in BMI, nutritional status and quality of life
**Tezacaftor/ivacaftor**	Phe508del-homozygous or Phe508del-heterozygous with a residual function mutation in *trans*, F508del heterozygotes with E56K, P67L, R74W, D110E, D110H, R117C, E193K, L206W, 711+3A→G, R347H, R352Q, A455E, D579G, E831X, 2789+5G→A, S945L, S977F, F1052V, K1060T, A1067T, R1070W, F1074L, 3272-26A→G, D1152H, D1270N, 3849+10kbC→T	Improved lung function, reduced pulmonary exacerbations, weight gain, improvement in quality of life
**Elexacaftor/tezacaftor/ivacaftor**	Phe508del-homozygous, Phe508del-heterozygous patients with a minimal function mutation in *trans*, Phe508del-any other mutation	Improved lung function gain (10–14%), reduced pulmonary exacerbations (by ∼63%), decrease in sweat chloride, improvement in quality of life

FEV_1_: forced expiratory volume in 1 s; BMI: body mass index. Information from [26, 27].

While the efficacy of CFTR modulators has been demonstrated in clinical studies, the partial restoration of CFTR function is likely to be suboptimal for normal physiological function and unlikely to significantly resolve chronic structural lung damage. Big challenges remain in finding new drugs that are even more efficacious, and therapies that will be effective for the remaining 10–15% of individuals who have mutations that are unresponsive to the small molecules currently available.

## CF and microbial infections

Recurrent and chronic respiratory bacterial infection is common in people with CF. Chronic infection with specific bacteria, frequency of infective exacerbations and recovery from exacerbations are associated with reduced lung function, increased morbidity and reduced survival [30]. During the early years of life, *S. aureus* and *H. influenzae* are the predominant microorganisms, while in adults, *P. aeruginosa* predominates [4, 5]. The adaptive mechanisms of *P. aeruginosa* help the bacterium to exist both in microcolonies and as biofilms in CF [31]. In addition, *P. aeruginosa* infections can evolve to a mucoid phenotype and elicit a major inflammatory response that results in accelerated lung function decline and is associated with lung transplantation or premature death [32–34]. Early *P. aeruginosa* infections usually have a low bacterial density, are environmentally acquired and can often be eradicated with systemic and/or inhaled antibiotics if identified early [35, 36]. The inhaled antibiotics are often used in combination with oral/*i.v.* antibiotics for the eradication of *P. aeruginosa*. Other microbes identified during the later stages of the disease include *Burkholderia cepacia* complex, *Stenotrophomonas maltophilia* and *Achromobacter xylosoxidans*, fungi including *Aspergillus* species and nontuberculous mycobacteria [4, 37].

With CFTR modulator treatment, there are still knowledge gaps in understanding the host–microbial interactions and their impact on airway physiology, infection and the patient's susceptibility to infection. However, the literature reveals contrasting reports on this topic. Some studies reported that the CFTR modulators may reduce bacterial load, microbial burden and restore innate immune responses and bacterial diversity similar to people without CF, thereby yielding an airway microbiome which reduces the incidence of acute airway infection and the rate of lung decline [38, 39]. Another study contradicts this by reporting that treatment with ivacaftor does not reduce the odds of culture positivity with common CF-related microorganisms such as *S. aureus* [39]. CFTR modulators have not demonstrated change in antibiotic susceptibility of microorganisms and have no impact on the control of viral infection in the CF airway epithelial cells [40–42]. With respect to the structural changes in lung, CFTR modulator therapy may not change or reverse the structural damage in the lungs caused due to CF. Imaging studies have shown improvements in mucous plugging in patients treated with ivacaftor and elexacaftor/tezacaftor/ivacaftor. However, it is too soon to determine whether the natural history of structural lung disease is altered [43–45].

### Infections in patients with CF

In people with CF, the diagnosis of respiratory infection is established by respiratory tract sample cultures, such as expectorated sputum (the preferred test sample), induced sputum, oropharyngeal swabs and cough swabs. In general, bronchoalveolar lavage is only undertaken for specific clinical indications or if the procedure is part of a clinical trial [46, 47]. Challenges associated with the diagnosis of infection in people with CF include availability of specimen, sensitivity of the diagnostic method and identification of multiple infections. As highly effective CFTR modulators reduce the volume of expectorated airway mucus, there is less availability of sputum samples to further culture and identify specific microorganisms. Obtaining samples is particularly challenging in children and adults with maintained lung function. While culture methods are the standard diagnostic procedure, serological methods and next-generation sequencing may help in identifying early colonisation and provide more information on the variety of infecting and colonising organisms in the lung microbiome [48].

### Challenges of *Pseudomonas aeruginosa*

Inhaled antibiotics are the standard of care for treating chronic infection with *P. aeruginosa* in people with CF to reduce *P. aeruginosa* density, host inflammation, maintain lung function and decrease the frequency of acute pulmonary exacerbations [49]. The identification of *P. aeruginosa* is straightforward if samples are available. In those receiving CFTR modulator therapy, sputum expectoration is often reduced significantly, with many individuals unable to provide routine samples for microbial culture and antimicrobial susceptibility tests. This makes it more difficult to identify the presence or absence of *P. aeruginosa* either chronically or during acute exacerbations. Additionally, it raises speculation regarding the definition of intermittent infection: whether it holds good in these patients, where there is less access to sputum samples. Determining and understanding the antimicrobial-resistant nature of the *P. aeruginosa* strain will help to optimise the treatment decision in people with CF.

## CF and treatment with inhaled antibiotics

Inhaled antibiotics such as aztreonam, colistin, levofloxacin and tobramycin are the mainstay of treatment for recurrent and chronic pulmonary infections caused by *P. aeruginosa* or as a suppressive therapy for other infections such as *Achromobacter* and *Stenotrophomonas*. Although they improve symptoms and reduce the frequency of pulmonary exacerbations, rarely people with CF may develop intolerance to inhaled antibiotics with reported side-effects such as bronchospasm, ototoxicity and acute kidney injury [50–52]. Oral azithromycin is used for a combination of antimicrobial and anti-inflammatory effects and is widely used to reduce exacerbations and improve quality of life [53]. Inhaled antibiotics are evidence-based and provide a practical approach to delivering high concentrations of drug to the airways while limiting systemic exposure. They have a proven efficacy in treating first and subsequent intermittent infections in people with CF [54]. They can be used with oral antibiotics to treat milder exacerbations, reduce frequency of exacerbations and avoid hospitalisation and the need for *i.v.* antibiotics. Administering aminoglycosides *via* the inhaled rather than the intravenous route reduces the risk of kidney damage and ototoxicity [19]. The efficacy and safety of aztreonam, colistin, levofloxacin and tobramycin in the management of CF are well established. They improve lung function and quality of life and reduce hospitalisations, concomitant intravenous therapies, number of exacerbations and bacterial load in people with CF [55].

Inhaled antibiotics are the standard of care for the eradication of new *P. aeruginosa* and recurrent/intermittent infections and reduce the concentration of *P. aeruginosa* in sputum and increase FEV_1_ as early as 2 weeks after initiation of treatment [36, 56]. The European CF Society guidelines recommend treatment with continuous suppressive therapy in people with CF and chronic *P. aeruginosa* infection (table 4) [6]. Early initiation of inhaled antibiotic therapy alone or in combination with oral antibiotics is an efficient strategy to delay chronic *P. aeruginosa* infections and reduce the decline in lung function, progressive lung damage and frequency of exacerbations [61].

**TABLE 4:** Guidelines that recommend inhaled antibiotics for the treatment of chronic *Pseudomonas* infections in people with cystic fibrosis (CF)

	**Recommendations**
**Standards for the clinical care of children and adults with cystic fibrosis in the UK [** **57** **]**	Inhaled antibiotics to be prescribed for long-term treatment of chronic *Pseudomonas aeruginosa* lung infections.
**National Institute for Health and Care Excellence guidelines [** **58** **]**	Eradication therapy with a course of oral or intravenous antibiotics, together with an inhaled antibiotic to be commenced for a person with CF developing a new *P. aeruginosa* infection. This would be followed with an extended course of oral and inhaled antibiotics. Sustained treatment with an inhaled antibiotic to be considered for people with CF who have chronic *Burkholderia cepacia* complex infection and declining pulmonary status, to suppress the infection.
**European CF Society best practice guidelines, 2018 [** **6** **]**	Long-term inhaled antibiotic therapy to be initiated for chronic bacterial infection with *P. aeruginosa*, when eradication therapy has failed.
**Cystic Fibrosis Foundation consensus guidelines for the care of individuals with advanced cystic fibrosis lung disease, 2020 [** **59** **]**	A trial of continuous alternating inhaled antibiotics (as dictated by bacterial pathogens identified in respiratory culture) is recommended for people with advanced CF lung disease.
**Cystic Fibrosis Standards of Care, Australia [** **60** **]**	Children with CF require regular inhaled therapy which includes bronchodilators, inhaled steroids, antibiotics, Pulmozyme and hypertonic saline.

However, inhaled antibiotics should be discontinued following successful eradication of an organism or when samples remain persistently negative following appropriate microbiological surveillance. Nationwide registries may serve as an important source of information on practice patterns and help to provide historical control populations for new therapies [62].

## Role of antibiotics amid CFTR modulators

CFTR modulator therapy is highly effective and is now part of standards of care for the majority of people with CF [61, 63]. Access to these drugs remains limited in several countries owing to their high cost. Furthermore, pulmonary exacerbations still occur in patients receiving CFTR modulator therapy, and there is a wide range of response observed in real-world evidence data and possibly a change in the symptom profile associated with exacerbations. Studies suggest that early initiation of modulator therapy (*i.e.* initiated at a younger age) might reduce the risk of lung infections [39]. However, there is no evidence so far that people with CF can stop or alter their current standard of care (antibiotics, physiotherapy, mucolytic agents, macrolides, *etc*.) while on CFTR modulators, especially since chronic infections are common in CF and may not disappear with current CFTR modulators.

There is some evidence to suggest that restoring CFTR function in the airways of people with CF may act synergistically with certain antibiotics owing to changes in airway surface liquid (ASL), pH, alterations in the microbiome, altered inflammatory and immune responses and increased killing through activation of innate molecules such as defensins [38]. Since modulators reduce the bacterial density, the antibiotic susceptibility of certain pathogens may be increased owing to the inoculum effect. The normalisation of ASL and mucus secretions result in increased mucociliary clearance, a process that may reduce the tolerance of microorganisms and decrease intrastrain genetic diversity [64].

Durfey *et al*. [65] reported that people with CF receiving ivacaftor, a highly effective modulator therapy, acquired fewer pathogens. Their study investigated whether combining ivacaftor with an intensive 3.5-month antibiotic course would clear chronic lung infections caused by *P. aeruginosa* or *S. aureus* in patients with R117H-CFTR, who are highly responsive to ivacaftor. The results showed that ivacaftor alone improved CFTR activity, lung function and inflammation within 48 h and achieved a ∼10-fold reduction in *P. aeruginosa* and *S. aureus* pathogen density within a week. While antibiotics produced an additional ∼10-fold reduction in pathogen density, this reduction was transient in patients who remained infected [64]. Data shows that while the bacterial density of *P. aeruginosa* decreased after the initiation of modulator therapy, it returned to pre-treatment values after 1 year of treatment [64, 66]. Furthermore, chronic infections may persist in modulator-treated patients, who might benefit from inhaled antibiotics to maintain long-term stability [67]. Clinical trials assessing the efficacy of CFTR modulators were conducted without adjusting concomitant medication, including inhaled antibiotics and mucolytics. Stopping such therapy would have been an exclusion criterion, but it is not clear that de-escalation of such therapies is advisable. There are two large, randomised studies in which mucoactive therapies will be de-escalated in people with CF treated with modulator therapies to determine whether the treatment burden can be reduced [68–70]. Reluctance from parents, people with CF and physicians to stop inhaled antibiotics is notable in the design phase of the study. Since both the studies focused on mucoactive treatments, it is unknown whether the treatment's effects observed in the studies will be only due to the CFTR modulators [68–70]. Additionally, some patients discontinue CFTR therapy or prefer on/off treatment with CFTR modulators rather than continuous therapy [70]. Furthermore, the global pandemic adds the risk of patients not being treated or patients not adhering to therapy in the new-normal scenario [71].

Despite CFTR-related improvements in wellbeing and FEV_1_, significant structural lung damage remains. Despite these changes, some people with CF receiving CFTR modulators can discontinue their antibiotics treatment once their FEV_1_ becomes stable and they feel relatively asymptomatic. This may potentially exacerbate chronic infection and be impactful in the longer term. Hence, long-term studies are required to assess the impact of infection on exacerbations and deterioration by monitoring lung function in combination with more sensitive tools such as lung clearance index, computed tomography and magnetic resonance imaging. Characterising infection status has become more difficult because sputum samples are less available due to the effect of CFTR modulators on mucus retention.

Inhaled therapy may be administered as continuous or alternate-month therapy. In some individuals, lung function may fall and symptoms increase during the month off therapy, necessitating continuous treatment. The presence and severity of underlying lung disease should be considered when making treatment decisions. Patients with severe lung disease, *e.g.* individuals on transplant waiting lists/with advanced CF lung disease may require continuous therapy with inhaled antibiotics. Hence, a personalised therapy would benefit the patients.

Further research on sensitive biomarkers and data from real-world-evidence studies and registries may help in deciding which therapies could be continued/discontinued during treatment with specific modulator therapy [25]. In future, real-world, multicentre, longitudinal, cohort studies (especially in young children) may be conducted for a relatively shorter period of time (~1 year) to understand the outcomes in CFTR modulators treated people with CF, after discontinuing antibiotics. However, defining the outcomes for this study may be a challenge. Efforts are needed to ensure that access to conventional evidence-based therapies is maintained worldwide without any insurance coverage issues. Future collaboration between all worldwide CF registries could lead to more insights in this area, and to enable such a situation, providing access to all CF treatments for all patients might be essential.

## Future directions

CFTR modulators have significantly changed CF treatment priorities. Inhaled antibiotics are still recommended for patients with chronic infections, as there are no long-term data to advocate discontinuation of antibiotic therapy for individuals on CFTR modulators. While taking treatment decisions, clinicians are encouraged to balance the simplification of treatment with the risk of clinical deterioration due to microbial infections. Hence, it is recommended that patients continue their existing medications while receiving CFTR modulators until more data on this topic are available. Further research on the long-term effects of CFTR modulators in people with CF with chronic infections might guide clinicians taking treatment decisions in routine practice.

**Points for clinical practice**

- Inhaled antibiotics continue to be prescribed for cystic fibrosis patients who receive cystic fibrosis transmembrane conductance regulator (CFTR) modulators to treat chronic respiratory infections.
- Patients are recommended to continue their existing treatment regimen while receiving CFTR modulators.
- Clinicians are encouraged to balance the simplification of treatment with the risk of clinical deterioration due to microbial infections when making treatment decisions.
